# Evaluation of Haemoglobin and Cytochrome Responses During Forearm Ischaemia Using Multi-wavelength Time Domain NIRS

**DOI:** 10.1007/978-3-319-55231-6_10

**Published:** 2017-03-21

**Authors:** Frédéric Lange, Luke Dunne, Ilias Tachtsidis

**Affiliations:** Department of Medical Physics and Biomedical Engineering, University College London, London, UK

**Keywords:** NIRS, TRS, Cytochrome-c-oxidase, Cuff Occlusion, Haemoglobin

## Abstract

We demonstrate the ability of a 16-wavelength time domain near-infrared spectroscopy system to monitor changes in oxy- and deoxy haemoglobin ([HbO_2_] [HHb]) and the oxidation of cytochrome-c-oxidase ([oxCCO]), during forearm ischaemia. We tested two methods to retrieve the concentration changes. The first uses the measured changes in light attenuation and the modified Beer-Lambert law, and the second uses the absorption and scattering estimated by the measured time-point spread function. The system is able to retrieve the concentration changes with both methods, giving similar results. At the end of forearm ischaemia (t = 5 min), we measured an increase in [HHb] of 16.77 ± 2.52 and 16.37 ± 2.33 μMol, and a decrease in [HbO_2_] of −6.12 ± 1.62 and −5.57 ± 2.02 μMol for method 1 and 2, respectively. At that same time, the changes in [oxCCO] were −0.36 ± 0.33 and −1.40 ± 1.20 μMol, for method 1 and 2, respectively. These small changes in [oxCCO], despite a huge change in haemoglobin, demonstrate the absence of crosstalk and are comparable to previous measurements using broadband NIRS.

## Introduction

Near-infrared spectroscopy (NIRS) is now a common tool for monitoring non-invasively the tissue in-vivo changes in oxy- and deoxy haemoglobin ([HbO_2_] [HHb]) [1], and has been extensively used to monitor the function and physiology of the brain [2–4].

Most of the Haemoglobin and cytochrome responses monitor changes in [HbO_2_], [HHb] using the modified Beer-Lambert law and assume a constant differential pathlength factor to account for the effect of the scattering in tissues [5]. These instruments are often referred to as continuous wave (CW) instruments and are highly portable, inexpensive, and easy to use. NIRS CW instruments measure the changes in light attenuation at two or three wavelengths; however, recent upgrades that utilize white light sources and broadband spectrometers for detection allow to measure changes in light attenuation at hundreds of wavelengths [6]. These broadband NIRS instruments can then be used to retrieve information on a third chromophore, the cytochrome-c-oxidase CCO. The Haemoglobin and cytochrome responses is the terminal electron acceptor of the respiratory chain in the mitochondria. NIRS monitors the changes in the redox state of the CCO using the oxidase minus reduced CCO spectra ([oxCCO]): Bale et al. provide a recent review on this topic [7]. Hence, the monitoring of the CCO provides information about metabolism and in particular mitochondrial oxygenation. This extra information, coupled with the classical vascular oxygenation measured by NIRS, enable us to study the link between them, which can be informative of the physiological status of the tissue. While CW NIRS instruments can only measure changes in light attenuation, time domain (TD) systems can quantify absorption and scattering. Haemoglobin and cytochrome responses can record the arrival time of photons that have traveled through tissue. Then, by fitting the measured arrival of the photon’s curve to an analytical model, the absorption and scattering coefficient can be estimated [8]. TD systems are less common because of their higher cost, complexity and size. However, improvement in the quality and decrease in the cost of components, such as fiber lasers and detectors, have increased the use of this technique [9]. One of the current developments of the TD systems is to perform measurements with a considerable number of wavelengths. The main benefit is to be able to retrieve the true optical properties of the tissues over a large bandwidth, which can then be used to estimate the tissue’s composition [10]. These systems can also be used to retrieve the CCO chromophore [11].

Towards that goal we have developed a multi-wavelength TD system, described in [12]. Here we demonstrate the ability of our multi-wavelength TD system to retrieve [HbO_2_], [HHb] and [oxCCO] responses, by replicating the forearm ischaemia experiment of Matcher et al. [12].

## Methods

The instrument is based on a supercontinuum laser coupled with an acousto-optical tunable filter that permits the selection of 16 narrow wavelengths, with a full width at half maximum between 2 and 4 nm, in the range of 650–1100 nm. The light is then transmitted on the tissue via a single core optical fibre. The system allows up to two sources points. On the detection side, four optical fibres collect the reflected light to four photon multiplier tubes (PMTs). Then, a router is used to redirect the signal to a single TCSPC card, in order to measure the arrival time of the photons.

Six healthy volunteers (age 24–29 years; 2 females) were recruited from the lab and informed consent was obtained. Measurements were performed on the medial aspect of the left arm. One source and four detectors were used in this experiment. The emitter and receiver fibres were separated from a relative distance ρ = 3.0 cm by a custom 3D printed probe holder. The 4 detectors were placed close together in a ring arrangement around the sources, and were probing the same area. Measurements were performed in a dark room to decrease the amount of background light. The volunteers positioned their arm in a comfortable resting position on a flat surface. A Haemoglobin and cytochrome responses was placed loosely around the left arm. After an initial 5-min period in resting position, the cuff was rapidly inflated to a pressure of 220 mmHg to provide an abrupt venous and arterial occlusion. The cuff occlusion was maintained for 5 min. The cuff was then released and monitoring continued for an additional 5 min. Time-resolved reflectance measurements were simultaneously performed at 16 wavelengths, from 780 to 870 nm, every 6 nm. The acquisition time for every wavelength was 50 ms, resulting in an acquisition frequency of about 1 Hz, including the dead times of the system. We tested two methods in order to retrieve the concentration changes in [HbO_2_], [HHb] and [oxCCO]. For method 1 we used the change in attenuation and the modified Beer-Lambert law. This method has the advantage of having a good signal-to-noise ratio (SNR). In order to account for the pathlength, we calculated the true pathlength for every wavelength using the mean arrival time of the photons recorded by the system [12]. Method 2 used the change of the absorption coefficient, obtained by fitting the solution to the diffusion equation for a semi-infinite homogenous medium to the Temporal Point Spread Function (TPSF) [7]. This method suffered from a lower SNR compared with method 1, but has the advantage of being able to distinguish between the change in absorption and scattering. To improve the SNR of method 2, we averaged the data across 10 time points. The data analysed with method 1 were not averaged because of the sufficient SNR with that technic.

Data are presented as mean and the standard error of mean (SEM).

## Results

**Fig. 10.1:**
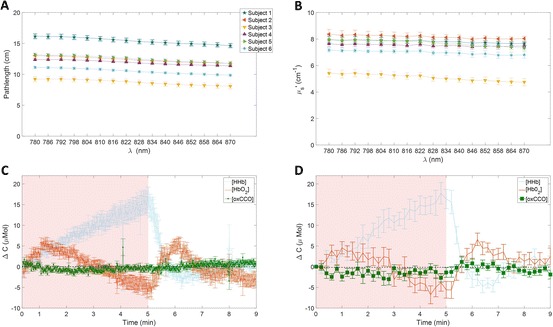
(**a**) Pathlength at rest of every channel for all subjects. (**b**) Scattering coefficient at rest of every channel for all subjects. (**c**) Grand average across all channels and all subjects (n = 24) of the concentration changes for [HHb], [HbO_2_] and [oxCCO] from method 1. (**d**) Grand average across all channels and all subjects (n = 24) of the concentration changes for [HHb], [HbO_2_] and [oxCCO] from method 2

Figure 10.1a shows the mean pathlength of the rest period for all participants, including all channels. It should be noted that, since all channels were probing the same area and similar results were found with each of them, we averaged the data across all the channels. The pathlength shows a strong variability among subjects with values (at 810 nm) ranging from 9.04 ± 0.41 cm for subject 3 to 15.76 ± 0.37 cm for subject 1. Figure 10.1b presents the mean scattering coefficient of the rest period for all subjects. The scattering coefficient shows a strong variability (at 810 nm) from 5.26 ± 0.26 cm^−1^ for subject 3 to 7.86 ± 0.22 cm^−1^ for subject 1. This variability might explain the variability of the pathlength. Indeed, the pathlength is dictated by the absorption and scattering coefficient of the tissues. Then, a change in at least one of those properties would have an influence on it [8]. Figure 10.1c presents the grand average across every channel and all subjects (n = 6 subjects × 4 detectors = 24) of the changes in concentration of [HbO_2_], [HHb] and [oxCCO] calculated with method 1. Figure 10.1d presents the same grand average with method 2. We see the typical response of haemoglobin to muscle ischaemia with a progressive increase of [HHb] and a progressive decrease of [HbO_2_], with the maximum change at the end of occlusion. Indeed, after 2.5 min of occlusion, the changes in [HHb] and [HbO_2_] calculated with methods 1 and 2, are 8.99 ± 1.02 and 9.48 ± 2.49 μMol, and 1.42 ± 0.97 and 0.75 ± 1.50 μMol, respectively. After 5 min, at the end of the occlusion, those changes have increased for [HHb] to 16.77 ± 2.52 and 16.37 ± 2.33 μMol, and for [HbO_2_] to −6.12 ± 1.62 and −5.57 ± 2.02 μMol, for methods 1 and 2, respectively.

The time course of the [oxCCO] is different to that of the haemoglobins. After 2.5 min of occlusion, the changes calculated with methods 1 and 2 are −1.03 ± 0.36 and −1.55 ± 1.13 μMol, respectively. After 5 min, at the end of the occlusion, those changes are −0.36 ± 0.33 and −1.40 ± 1.20 μMol, respectively. Moreover, both methods of chromophore estimation show a similar qualitative behaviour and a very good agreement in terms of magnitude. These results are summarised in Table 10.1.

**Table 10.1:** Main results of the study: mean values with the standard error of the mean, calculated with both methods, of [HHb], [HbO_2_], and [oxCCO] after 2.5 and 5 min of occlusion

	2.5 min after occlusion			5 min after occlusion		
	[HbO_2_] (μMol)	[HHb] (μMol)	[oxCCO] (μMol)	[HbO_2_] (μMol)	[HHb] (μMol)	[oxCCO] (μMol)
MBLL	1.42 (±0.97)	8.99 (±1.02)	−1.03 (±0.36)	−6.12 (±1.62)	16.77 (±2.52)	−0.36 (±0.33)
Δμ_a_	0.75 (±1.50)	9.48 (±2.49)	−1.55 (±1.13)	−5.57 (±2.02)	16.37 (±2.33)	−1.40 (±1.20)

*MBLL* modified Beer-Lambert law

## Discussion

This study shows that, during cuff occlusion, there is an increase in [HHb] and a decrease in [HbO_2_], with the maximum change occurring at the end of occlusion. The [oxCCO] showed a different dynamic response compared with the haemoglobins. Matcher et al. [12] postulated that the changes in [oxCCO] during muscle ischaemia should be small. This behaviour was observed in the present study and our results are in good agreement with the literature [12].

We observed that the [HbO_2_] response at 2.5 min (middle of the occlusion) was positive. This behaviour is expected since cuff inflation initially occludes the veins, then the arteries. Thus, it causes an augmentation of the [HbO_2_] at the beginning of the occlusion before the decrease (Fig. 10.1c, d).

Both methods used for estimation of the chromophore changes showed the same behaviour with similar magnitude changes, especially for [HHb] and [HbO_2_]. This agreement might be because we used the true pathlength to calculate the changes of the chromophores with method 1.

The small change of the [oxCCO] is a good indication of the absence of crosstalk between haemoglobin and cytochrome, which is one of the major issues when trying to resolve the [oxCCO] [4]. Indeed, in the presence of crosstalk, the changes in magnitude of the [oxCCO] during a muscular cuff occlusion can be the same as that of the haemoglobins [12].

Both methods qualitatively demonstrate a difference in the dynamic response between [oxCCO] and haemoglobin during a Haemoglobin and cytochrome responses (Fig. 10.1c, d). In method 1, the average [oxCCO] change, at the end of the occlusion, is very small compared with changes in the hemoglobins. However, in method 2, this difference between haemoglobins and [oxCCO] response was smaller. It is worth noting that we averaged the data in order to extract information with method 2, due to the lower SNR of that method. Despite that fact, Table 10.1 and Fig. 10.1 show that the concentration changes retrieved with method 2 are noisier. This might explain the discrepancy between the two methods for the [oxCCO] quantification. Therefore, we are investigating improvement in methods and instrumentation to overcome this issue.

Method 2 is able to retrieve the concentration changes of the three Haemoglobin and cytochrome responses with good agreement with method 1. Thus, using our system and method 2, we can investigate the effect of scattering and extract the absolute concentration. The variation of the scattering coefficient could influence the measurement of a small chromophore like CCO; this effect will be investigated in the future.

In conclusion, this Haemoglobin and cytochrome responses is able to distinguish between the haemoglobin and cytochrome signal, without crosstalk, during muscle ischemia. It is also shown that our system is able to retrieve the absorption and scattering coefficient of muscle tissue.
